# Immunogenicity of the *Plasmodium vivax* merozoite surface protein 1 paralog in the induction of naturally acquired antibody and memory B cell responses

**DOI:** 10.1186/s12936-017-2000-z

**Published:** 2017-08-30

**Authors:** Hay Man Kyaw Min, Siriruk Changrob, Phyu Thwe Soe, Jin Hee Han, Fauzi Muh, Seong-Kyun Lee, Patchanee Chootong, Eun-Taek Han

**Affiliations:** 1 Department of Clinical Microbiology and Applied Technology, Faculty of Medical Technology, Mahidol University, Bangkok, 10700 Thailand; 2 Department of Medical Environmental Biology and Tropical Medicine, School of Medicine, Kangwon National University, Chuncheon, Gangwon-do, 200-701 Republic of Korea

**Keywords:** *Plasmodium vivax*, Merozoite surface protein 1 paralog, Immunogenicity

## Abstract

**Background:**

The *Plasmodium vivax* merozoite surface protein 1 paralog (PvMSP1P-19) is a glycosylphosphatidylinositol (GPI)-anchored blood-stage protein that is expressed on the merozoite surface. It is proposed as a blood-stage vaccine candidate against *P. vivax* because of its ability to induce immune responses upon natural *P. vivax* exposure and in immunized animals. This study aimed to demonstrate the presence of inhibitory antibodies and memory B cell responses to the PvMSP1P-19 antigen during acute *P. vivax* infection and after recovery from infection.

**Methods:**

To evaluate the antibody responses to PvMSP1P-19 during and after recovery from *P. vivax* infection, heparinized blood was collected from *P. vivax*-infected patients and recovered subjects to detect the total IgG response. The seropositive samples were defined into high and low responders, according to their optical density (OD) values obtained from ELISA. High responders were the subjects who had OD values above the OD of antisera from non-exposed controls plus 4× standard deviations, whereas low responders were the subjects who had OD values less than OD of antisera from non-exposed controls plus 4× standard deviations. The plasma from high and low responders were taken for testing the inhibitory activity against PvMSP1P-19-erythrocyte binding by in vitro EBIA. The sustainability of PvMSP1P-19-specific memory B cell responses after recovery from infection was analysed by ELISPOT.

**Results:**

The anti-PvMSP1P-19 antibody levels were significantly higher in acutely infected *P. vivax* patients compared to healthy controls (*P* <  0.0001). Monitoring of the anti-PvMSP1P-19 antibody titre showed that the antibody was maintained for up to 9 months after recovery. Almost all high-responder groups strongly inhibited PvMSP1P-19 binding to erythrocytes, whereas no inhibition was shown in most low-responder samples. Interestingly, the inhibitory activity of the antibodies in some individuals from high-responder samples were stable for at least 12 months. The longevity of the antibody response was associated with the presence of PvMSP1P-19-specific memory B cells at 9 months after recovery from infection.

**Conclusions:**

The PvMSP1P-19 antigen has immunogenicity during the induction of the antibody response, in which both the levels and inhibitory activity are maintained after the patient recovered from *P. vivax* infection. The maintenance of the antibody response was associated with the response of PvMSP1P-19-specific memory B cells. Therefore, the PvMSP1P-19 antigen should also be considered as a reliable vaccine candidate to develop a blood-stage vaccine against *P. vivax*.

## Background

*Plasmodium vivax*, the most frequent cause of recurring malaria, is widely distributed throughout Latin America, some parts of Africa and Southeast Asian countries [1–3]. An estimated 2.85 billion people worldwide are at risk of vivax malaria and approximately 65% of cases are from Asian and South American regions [4]. The major features of *P. vivax* that distinguish it from other *Plasmodium* species are its high transmission potential from early and continuous gametocyte production, its hibernating behaviour in the liver in the form of hypnozoites, its shorter life cycle and its high infectivity in vectors, which are the primary obstacles to controlling this type of malaria [5]. In this regard, the development of a vaccine to attack vivax malaria is urgently needed to reduce morbidity and mortality related to malaria. However, due to technical limitations such as lack of continuous culture, the development of effective vivax vaccine has been delayed, and the eradication of vivax malaria is still challenging.

Among three types of vaccine candidates-pre-erythrocytic, erythrocytic and transmission blocking-the erythrocytic approach is targeted primarily toward minimizing the morbidity, mortality and parasitaemia levels. Blood stage antigens such as apical membrane protein 1 (AMA-1), merozoite surface protein 1 (MSP-1) and Duffy-binding protein (DBP) are major targets for blood-stage vaccine development because they are responsible for the clinical manifestation and merozoite invasion of red blood cells in humans [6]. It is possible to acquire immunity against blood stage antigens in natural exposure-derived *P. vivax* infections. Similarly, protection against *P. vivax* infection could be induced by numerous immunization strategies [7]. However, the rapid mutation of the blood-stage antigens is the primary challenging problem in its vaccine development. In fact, an effective vaccine must be able to induce both humoral and cellular immune responses without having any genetic restriction while stimulating memory cells. Therefore, the discovery of potential vaccine antigens and a better understanding of the underlying immune mechanisms against parasites in natural infections will guide us to progress further in developing this vaccine.

Recently, a new vaccine candidate has emerged for blood stage vivax malaria infections called *Plasmodium vivax* merozoite surface protein 1 paralog (PvMSP1P-19), which is located at the upstream locus of the MSP1 gene. It was identified as a paralog of PvMSP1, and it showed similarities in the size, molecular mass, number and location of cystine residue, whereas it was not expressed as a highly polymorphic protein as was PvMSP1 [8, 9]. Interestingly, PvMSP1P-19 was found to contain double epidermal growth factor (EGF)-like domains, which assist in merozoite invasions of erythrocytes. As a consequence, the rosette forms of PvMSP1P-19-transfected COS7 cells and human erythrocyte binding observed during in vitro study was stronger than those seen in PvMSP1-infected cells. This result assures us that the function of PvMSP1P-19 is to play a role as a parasite ligand for invasion. Additionally, another advantage of PvMSP1P-19 is that both the N- and C-terminal regions have high potential for inducing host immune responses, and the resulting antibodies inhibit PvMSP1P-19-erythrocyte binding [10]. Another study also reported that PvMSP1P-19 strongly induces the activation of IFN-γ-producing effector cells following natural *P. vivax* exposure, suggesting that it has the potential to activate the recall response of Th1 effector memory cells [11]. These data all suggested that PvMSP1P-19 is reliable at inducing the inhibitory antibody and at promoting good immunogenicity. However, to be sure that PvMSP1P-19 antigen has the ability as a vaccine candidate, further studies are needed. Hence, in this study, the longevity of a naturally acquired antibody against the PvMSP1P-19 antigen was evaluated because it would be an important factor for determining the qualifications of the vaccine. Another objective of this study is to observe the specific memory B cell (MBC) response to PvMSP1P-19 in *P. vivax*-infected patients to acquire a better understanding of the underlying immune mechanisms and to develop an effective malaria vaccine in the future.

## Methods

### Study area and sample collection

To observe the antigenicity of PvMSP1P-19 antigen during acute and convalescence phase and the maintenance of anti-PvMSP1P-19 responses after recovery from *P. vivax* infection, both cross-sectional survey and longitudinal cohort study were performed between May 2014–October 2016 in a low-malaria-transmission area, Rap Ro malaria clinic, Chumphon Province, which is located in the southern part of Thailand where both *P. vivax* and *Plasmodium falciparum* commonly occur.

The antigenicity of PvMSP1P-19 in induction of antibody response in natural infection at acute malaria infection and after recovery from infection was explored. Ten milliliters of heparinized peripheral blood samples were collected from *P. vivax* acutely infected patients (n = 40) and from *P. vivax* subjects who had recovered from the infection at 3 months (n = 27), 9 months (n = 15) and 12 months (n = 14). In addition, the malaria endemic villagers (n = 15) who lived in the same village as acutely infected patients for more than 5 years showed negative parasitaemia in blood smears at the time of collection as well as have no history of malaria infection was recruited in this survey. The ages of subjects ranged from 18 to 63 years old.

The maintenance of anti-PvMSP1P-19 response after recovery from infection was demonstrated from a longitudinal analysis. Among 40 *P. vivax* infected patients, 16 infected individuals were followed antibody response at acute phase and after they recovered from infection for 3 months. Moreover, five infected individuals were followed anti-PvMSP1P-19 responses after 3, 9 and 12 months recovery from infection. Giemsa staining by thick and thin peripheral blood films was used for diagnosis, and nested PCR was performed to confirm the *P. vivax* infection. Individual samples were taken for plasma preparation and used for enzyme-linked immunosorbent assays (ELISA) and erythrocyte binding inhibition assays (EBIA).

The presence of memory B cell responses to the PvMSP1P-19 antigen after recovery from *P. vivax* infection was demonstrated by collecting peripheral blood mononuclear cell (PBMC) samples (n = 11) from individuals during acute phase and after 9 months recovery from infection with *P. vivax* between June 2016 and March 2017. Blood samples from healthy persons (n = 22) from the Faculty of Medical Technology, Mahidol University who had never been exposed to malaria in their lives were collected as healthy controls for this study. The study was approved by the Committee on Human Rights Related to Human Experimentation, Mahidol University, and the Ministry of Health, Thailand (MUIR 2012/079.2408).

### Recombinant PvMSP1P-19 protein expression

The recombinant protein PvMSP1P-19 was expressed and purified as previously described [10]. Briefly, the gene fragment encoding PvMSP1P-19, which was obtained from the genomic DNA of the *P. vivax Sal*I strain sequence, was amplified by PCR and cloned into the *Xho*I and *Not*I sites of the pEU-E01-His-TEV-MCS vector (Cell Free Sciences, Matsuyama, Japan). An ABI PRISM 310 Genetic Analyzer and a BigDye Terminator v1.1 Cycle Sequencing kit (Applied Biosystems, Foster City, CA, USA) were used to confirm the inserted nucleotide sequence. For the in vitro transcription and subsequent translation, highly purified plasmid DNA was prepared by using a Maxi Plus™ Ultrapure plasmid extraction system (Viogene, Taipei, Taiwan) in accordance with the manufacturer’s instructions. For recombinant protein expression, the purified DNA was eluted in 0.1× TE buffer (10 mM Tris–HCl, pH 8.0, 1 mM EDTA) and used in a wheat germ cell-free (WGCF) system (CellFree Sciences) as described previously [12, 13]. After that, the newly synthesized recombinant PvMSP1P-19-19 protein was purified using a nickel-nitrilotriacetic acid (Ni–NTA) column (Qiagen, Hilden, Germany).

### Detection of total IgG responses to PvMSP1P-19

The levels of the antibody response against PvMSP1P-19 in individuals with *P. vivax* exposure were measured by indirect ELISA. In brief, 5 μg/mL of recombinant PvMSP1P-19 protein was used to coat 96-well ELISA plates overnight at 4 °C. Then, 100 μL of 5% skim milk was added to each well and incubated for 2 h. Next, the plates were washed twice with washing buffer (0.05% Tween-20 in 1× PBS), and 50 μL of 1:200 diluted plasma was added to specific wells of the coated plate and incubated for 1 h. After that, the wells were washed again, followed by the addition of 50 μL of affinity-purified peroxidase-labelled goat anti-human IgG (H+L) antibody, which was diluted 1:1000 with blocking buffer. After 90 min of incubation at room temperature, another washing step was needed. After that, 50 μL of 2,2′-azino-bis(3-ethylbenzothiazoline-6-sulphonic acid) (ABTS) substrate solution was added. The ELISA plates were incubated for 45 min in the dark at room temperature. Finally, the optical density (OD) of each sample well was measured with an ELISA plate reader (SYNERGY™ HTX multi-mode reader, BioTek) at 405 nm (OD_405_). All the samples were analysed in duplicate and mean values were used in the analyses. The cut-off value was obtained from the mean plus two standard deviations (SDs) of the OD of all of the non-exposed Thai plasma samples. Based on ELISA data, the seropositive samples were classified into the following two groups: high responders (OD ≥ mean + 4SD of non-exposed Thai control plasma; 0.039–0.137) and low responders (OD < mean + 4SD of non-exposed Thai control plasma; 0.022–0.037).

### Erythrocyte binding inhibition assay of anti-PvMSP1P-19 antibody

In this study, human embryonic kidney 293 cells carrying the SV40 large T antigen (HEK293-T) were used in an EBIA specific to PvMSP1P-19. The cells were grown in Dulbecco’s modified Eagle medium (DMEM) (Gibco, Invitrogen, Paisley, UK) with 10% fetal bovine serum (FBS) (Gibco) at 37 °C with 5% atmospheric CO_2_. The cells were passaged when they reached 90–100% confluence. A 2 × 10^4^ concentration of cells was transferred into each well of a 24-well culture plate (Corning Inc., Corning, NY, USA). Next, the cells were transfected with 50 μL of DNA-liposome complex, and 100 ng of diluted plasmid pEGFP-MSP1P was mixed with 2% Lipofectamine^®^2000 (Invitrogen, Carlsbad, CA, USA) and incubated at 37 °C in a 5% CO_2_ chamber for 42–44 h. The transfection efficiencies were determined by observing the expression of green fluorescence protein (GFP) under an inverted fluorescence microscopy. The HEK293-T cells with transfection efficiencies of >80% were used for the EBIA.

To test the inhibition of the antibodies, 250 μL of 1:10 diluted plasma was added to transfected HEK293-T cells containing culture plate wells and incubated for 1 h at 37 °C. Then, 50 μL of 10% Duffy-positive human packed red blood cells was added to each well. The plates were incubated for 2 h, followed by the removal of the non-adherent erythrocytes by washing them with washing buffer (1× PBS with Mg^2+^ and Ca^2+^). Rosette-forming transfected HEK293-T cells in which adherent erythrocytes covered >50% of the HEK293-T cell surface were quantified under the 20× magnified objective lens of an inverted microscope (Olympus, CKX41, UK) for 30 fields per well. The plasma samples obtained from non-malaria naive individuals were considered negative controls, and mouse monoclonal antibodies against PvMSP1P-19 were used as the positive control. The percent binding-inhibition of each subjects was determined by assessing the number of rosettes in wells of transfected HEK293-T cells in the presence of *P. vivax* human plasma relative to rosettes in wells of transfected cells in presence of non-exposed Thai control plasma. Individual samples were dispensed into duplicate wells, and the whole experiment was repeated three times.

### Preparation of peripheral blood mononuclear cells

To demonstrate the presence of MBCs specific to the PvMSP1P-19 antigen, PBMC samples from individuals who had recovered from *P. vivax* infection for 9 months (n = 11) were taken for MBC phenotyping and ELISPOT assays. The PBMCs were prepared from heparinized blood, for which the blood was diluted with incomplete RPMI 1640 medium (Gibco, Invitrogen, Paisley, UK) at a 1:1 proportion. To separate the PBMCs, diluted blood was overlaid on Ficoll-Hypaque (Stemcell Technologies, Vancouver, Canada). The overlaid blood sample was centrifuged for 30 min at 2000 rpm and 18 °C. The lymphocytes were harvested and washed twice at 1500 rpm for 10 min at 4 °C, then resuspended in R10 medium (incomplete RPMI media supplement with 10% FBS and 100 units/mL penicillin, with 100 μg/mL streptomycin). Later, the cell counting process and cell viability evaluation were performed by Trypan blue exclusion test.

### Phenotyping memory B cells

To phenotype the MBCs, approximately 1 × 10^6^ PBMCs were stained with a panel of fluorochrome-conjugated monoclonal antibodies that consisted of anti-human CD19-FITC (Biolegend^®^, San Diego, CA, USA), anti-human CD10-PE Cy7 (Biolegend^®^) and anti-human CD27-PE (Biolegend^®^) and incubated for 15 min at 4 °C. The cells were washed with 500 μL of FACS buffer at 1500 rpm for 5 min at 4 °C. Then, the cells were resuspended in 250 μL of FACS buffer. Finally, the stained cells were phenotyped by flow cytometry (BD FACSCanto™; Becton–Dickinson, Oxford, UK) and the resulting data were analysed by FlowJo (v. 7.0; Tree Star Inc., San Carlos, CA, USA).

### PBMCs from stimulation for the ELISPOT assay

Before the ELISPOT assay was performed, PBMCs were first stimulated with polyclonal activator R848 and recombinant human IL-2 (Mabtech AB, Stockholm University, Sweden). During the cell stimulation process, 1 × 10^6^ cells per mL were seeded into the 24-well culture plate (Corning Inc.). In parallel to the stimulated samples, non-stimulated samples were also employed. All the samples were incubated at 37 °C in a humidified 5% CO_2_ incubator for 72 h.

### ELISPOT assay

MultiScreen Filter PVDF Immobilon plates (Merck Millipore, Darmstadt, Germany) were treated with 35% ethanol for 1 min prior to being washed five times with sterile water. The wells were then coated overnight at 4 °C with 15 µg/mL of monoclonal antibodies of anti-human IgG (clones MT91/145; Mabtech), 10 µg/mL of recombinant PvMSP1P-19 or PvMSP1 antigen, or 5 µg/mL of tetanus toxoid (TT) antigen (Merck Millipore, Darmstadt, Germany) diluted in sterile PBS. The plates were washed with 200 µL of sterile PBS five times and blocked with 200 µL of R10 for at least 30 min at 37 °C in 5% CO_2_. Both the stimulated and non-stimulated cells were harvested from the culture plates, counted and seeded in duplicate to yield 5 × 10^4^ cells per anti-human IgG-coated well and 1 × 10^6^ cells per specific antigen-coated well. After 24 h of incubation at 37 °C and 5% CO_2_, the plates were then washed with PBS five times. Then, 1 µg/mL of detection monoclonal antibodies MT78/145 (Mabtech) diluted in PBS-0.5% FBS were added and incubated for 2 h at room temperature. After five washes with PBS, the immobilized IgG was labelled using streptavidin–horseradish peroxidase (HRP)-conjugated polyclonal goat anti-human IgG (Mabtech) diluted to 1:1000 in PBS-0.5% FBS; Mabtech) and incubated for 1 h at room temperature. Following a thorough washing with PBS, tetramethylbenzidine (TMB) substrate (Mabtech) was added. The filtered plates were rinsed with deionized water after distinct spots emerged. Antigen-specific MBCs were expressed as spot-forming cells (SFC) in the wells. The plates were then left to dry and stored to protect them from light until they could be analysed with a Bioreader 5000 Pro-F gamma ELISPOT Reader (BioSys GmbH, Karben, Germany). PBMCs from *P. vivax* subjects that were cultured without stimulation and then incubated overnight with PvMSP1P-19 antigens were used as negative controls. The positive ELISPOT response was defined when spots were detected in duplicate wells and the total spots in the specific antigen-coated well reached at least twice the number of spots detected in the negative controls.

### Statistical analysis

The data were analysed using GraphPad Prism software (Systat Software, San Jose, CA, USA) and Microsoft Excel 2007. The nonparametric Mann–Whitney *U* test was used to determine the significant difference of the antibody titre level against PvMSP1P-19 antigen in *P. vivax* malaria-exposed subjects, endemic villagers and healthy controls. An unpaired *t*-test was used to measure the significant difference between the acute phase and after 3 months recovery from infection groups from same individual. The CD19^+^ total B cells and MBC frequencies between different time points in the same individual were compared by paired *t*-test. MBCs specific to PvMSP1P-19 antigens and TT antigens between stimulated PBMCs and the non-stimulated negative control were compared by using a Wilcoxon matched-pairs signed rank test. In all the analyses, *P* values of less than 0.05 (*P* <  0.05) were deemed to indicate statistical significance.

## Results

### Naturally acquired IgG responses to PvMSP1P-19

**Fig. 1:**
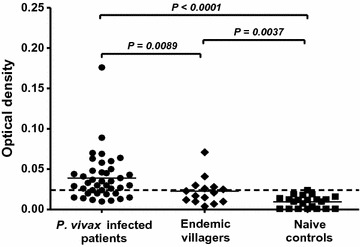
Humoral immune response to PvMSP1P-19. The antibody levels were detected in human plasma from acutely *P. vivax*-infected patients (n = 40), villagers in endemic areas (n = 15) and naïve controls (n = 22). Each symbol represents an individual sample. The *dashed line* indicates the cut-off value [mean ± 2 standard deviations (SD) of the optical density (OD) value of malaria-naïve individuals]. A nonparametric Mann–Whitney *U* test was used to assess the significant difference in the antibody titre levels against the PvMSP1P-19 antigen. The level of significance was set at a *P* value <0.05

To evaluate the serological response against the PvMSP1P-19 antigen in natural *P. vivax* exposure, the antibody levels in plasma from acute *P. vivax* infection, endemic villagers and healthy controls were determined. The result showed that there was a significantly higher antibody level in acutely *P. vivax*-infected subjects in relation to the healthy controls (average OD values: *P. vivax* patients = 0.039, healthy controls = 0.010, *P* < 0.0001) as well as significant differences in endemic villagers relative to the healthy controls (endemic villagers = 0.023, healthy controls = 0.010, *P* = 0.0037). Moreover, the antibody titre in acute patients was higher than that of endemic villagers (*P. vivax* patients = 0.039, endemic villagers = 0.023, *P* = 0.0089) (Fig. 1). According to the antibody titres in the individuals, 29 patients (72.5%) were seropositive for PvMSP1P-19. The seropositive individuals were classified as high responder (n = 16, OD > 0.039) and low responder (n = 13, OD < 0.037) groups (Fig. 1). There were six individuals (40%) from endemic villagers who produced antibodies to PvMSP1P-19 antigen. These data suggest that the PvMSP1P-19 antigen has immunogenicity and induces antibody production during natural infection.

### Longevity of anti-PvMSP1P-19 antibody responses

**Fig. 2:**
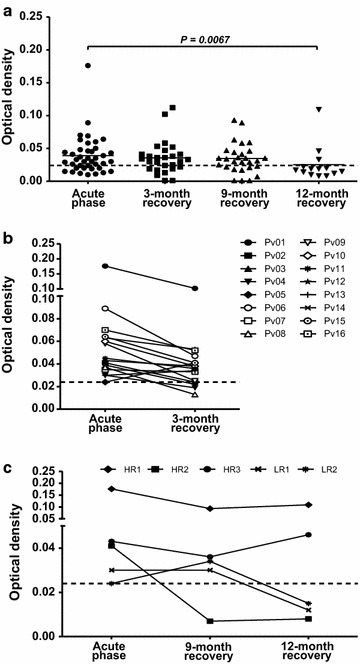
Longevity of the antibody response to the PvMSP1P-19 antigen after recovery from infection. **a** A survey of antibody responses during the acute phase (n = 40) and after anti-malarial treatment at 3 months (n = 27), 9 months (n = 28) and 12 months (n = 14). The *bars* represent the mean values. Mann–Whitney *U* test was used for statistical analysis and *P* value calculation. The level of significance was set at a *P* value <0.05. **b** The stability of the anti-PvMSP1P-19 antibody responses in the same individual (n = 16) during acute phase and at 3-month recovery phase. **c** A longitudinal cohort analysis of anti-PvMSP1P-19 response persistence in seropositive individuals (n = 5, HR1 = Pv01, HR2 = Pv02, HR3 = Pv03 , LR1 = Pv04, LR2 = Pv05) at their acute phases and by following-up their recovery phases at 3, 9 and 12 months. The *dashed line* indicates the cut-off value (means ± 2SD of the OD value of malaria-naïve individuals)

A follow up study of anti-PvMSP1P-19 responses in individual patients during acute phase and the recovery period at 3, 9 and 12 months showed that 72.5, 70.4, 67.9 and 21.4% were seropositive for PvMSP1P-19, respectively (Table 1). There were no significant differences in the levels of antibodies between subjects who had been recovered from infection at 3 months and 9 months compared to the acute infected patients (*P* = 0.6681 and *P* = 0.7265, respectively) (Fig. 2a). However, at 12 months after recovery from infection, the antibody levels were significantly reduced compared to the acutely infected patients (*P* = 0.0067) (Fig. 2a).

**Table 1:** A mass blood survey of the antibody responses to PvMSP1P-19 during acute phase and after recovery from *P. vivax* infection

Time point^a^	Total (*n*)^b^	No. positive (%)^c^	OD values					Significance
			Min^d^	Max^e^	Median^f^	SD^g^	*P* value^h^	
Acute phase	40	29 (72.5)	0.010	0.176	0.032	0.029	<0.0001	Acute vs healthy controls
3 months recovery	27	19 (70.4)	0.001	0.112	0.033	0.025	0.6681	Acute vs 3 months recovery
9 months recovery	28	19 (67.9)	0.001	0.093	0.032	0.023	0.7265	Acute vs 9 months recovery
12 months recovery	14	3 (21.4)	0.008	0.109	0.018	0.026	0.0067	Acute vs 12 months recovery

^a^ *Time point* the times at which the *P. vivax*-infected blood samples were collected

^b^ *Total* (*n*) the total numbers of samples collected at each time point

^c^ *Number of positive* (*%*) the number of seropositive individuals who had OD values greater than the cut-off value (mean ± 2 SD of the OD value of malaria-naïve individuals)

^d^ *Min* the lowest antibody level at each time point

^e^ *Max* the highest antibody level at each time point

^f^ *Median* the median OD value of antibody levels at each time point

^g^ *SD* the standard deviation of the antibody levels at each time point

^h^ *P value P* value of the difference between the mean antibody levels of *P. vivax*-infected subjects and malaria-naïve subjects that were compared using the Mann–Whitney *U* test

To explore the maintenance of antibody responses to PvMSP1P-19 during convalescence, the persistence of anti-PvMSP1P-19 antibody titre in individuals was observed after recovered from infection at 3 months. 81% of the patients had persistent antibody responses at 3 months after recovery. There was no significant difference in the antibody titres between samples from acute patients and those at 3 months after recovery from infection (*P* = 0.0966, Fig. 2b). Thus, for the further analysis of the antibody response persistence, 5 individual patients (HR1, HR2, HR3, LR1 and LR2) were followed for their antibody titres up to 12 months after recovery from the infection. The results showed that the anti-PvMSP1P-19 responses in HR1 and HR3 were maintained at a seropositive level for up to 12 months after recovery from the infection, whereas the antibody titre in HR2 was seronegative at 9 months after treatment. In addition, the LR1 and LR2 were seronegative at 9 months after recovery from infection (Fig. 2c). These analyses suggest that antibody response to PvMSP1P-19 are stably maintained after recovery from infection.

### The inhibitory activity of anti-PvMSP1P-19 antibody against erythrocyte binding

**Fig. 3:**
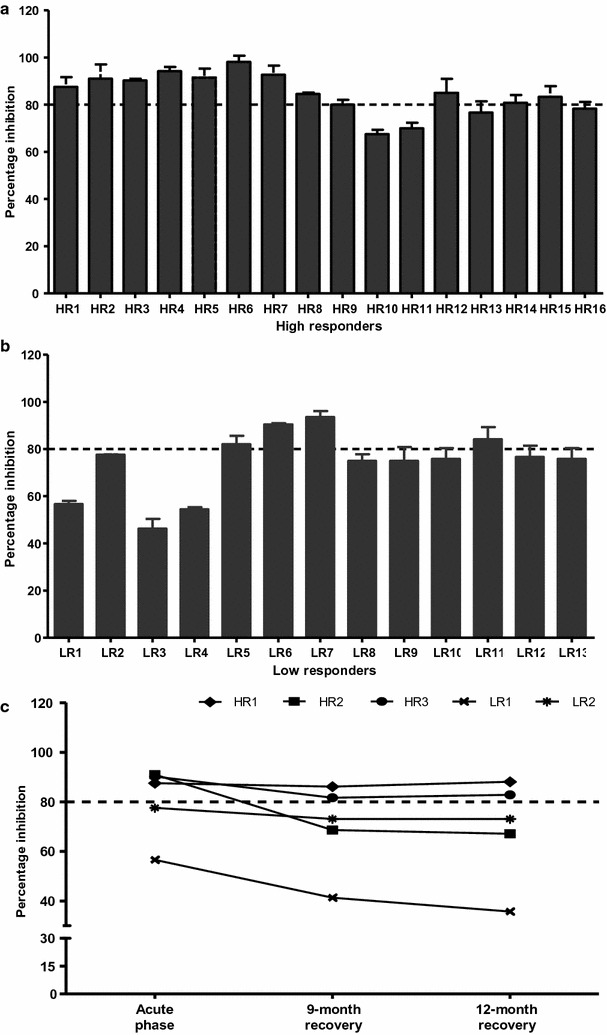
Inhibitory activity of antibodies against PvMSP1P-19 binding to human erythrocytes. Seropositive plasma samples from acutely *P. vivax*-infected **a** high responders (n = 16) and **b** low responders (n = 13) were performed to evaluate the inhibitory activity of antibodies against PvMSP1P-19-erythrocyte binding. **c** The activity of the anti-PvMSP1P-19 inhibitory antibody against human erythrocyte binding in individuals (n = 5) in their acute phases and their recovery phases at 9 and 12 months after *P. vivax* infeciton. The transfected HEK293-T cells expressing PvMSP1P-19 were incubated with plasma and with human erythrocytes. The numbers of rosettes were compared between wells of transfected cells that were incubated with plasma of vivax malaria patients relative to the negative control well. The charts show the mean inhibition of each *P. vi*vax subject. *Error bars* represent ± standard deviation

To evaluate the inhibitory efficiency of the anti-PvMSP1P-19 antibodies, plasma samples from high responders and low responders among the acutely *P. vivax*-infected subjects (n = 29) were taken for in vitro EBIA. Among the 16 high responders, 12 individuals produced high levels of inhibitory antibodies against PvMSP1P-19 binding to erythrocytes, at >80% inhibition activity, whereas the antibodies from 4 patient samples (HR10, HR11, HR13 and HR16) exhibited low inhibitory activity (Fig. 3a). In low responders (n = 13), or almost all the patients, 9 samples had a low ability to inhibit PvMSP1P-19-erythrocyte binding, at <80% inhibition activity. Only 4 patients strongly blocked human erythrocyte binding (LR5 = 82.1%, LR6 = 90.4%, LR7 = 93.6% and LR11 = 84.2%) (Fig. 3b).

In addition, the longevity of anti-PvMSP1P-19 antibodies against erythrocyte binding after anti-malarial treatment was observed from the HR and LR groups (n = 5) by following up with their acute and recovery phases after infection. The activity of inhibitory antibodies against PvMSP1P-19-erythrocyte binding in HR1 and HR3 was maintained by showing the inhibition percentages >80% at the acute and recovery phases. By contrast, the function of inhibitory antibodies in HR2 was not stable, and it showed a significant reduction at 9 and 12 months after recovery from infection (acute phase = 91.0%, 9 months recovery phase = 68.7% and 12 months recovery phase = 67.2%). In low responders, the antibodies in LR1 and LR2 weakly inhibited PvMSP1P-19-erythrocyte binding at the acute phase, and this process was continuously reduced during the convalescence period (Fig. 3c).

### Memory B cell surface analysis during acute phase and after recovery from infection

**Fig. 4:**
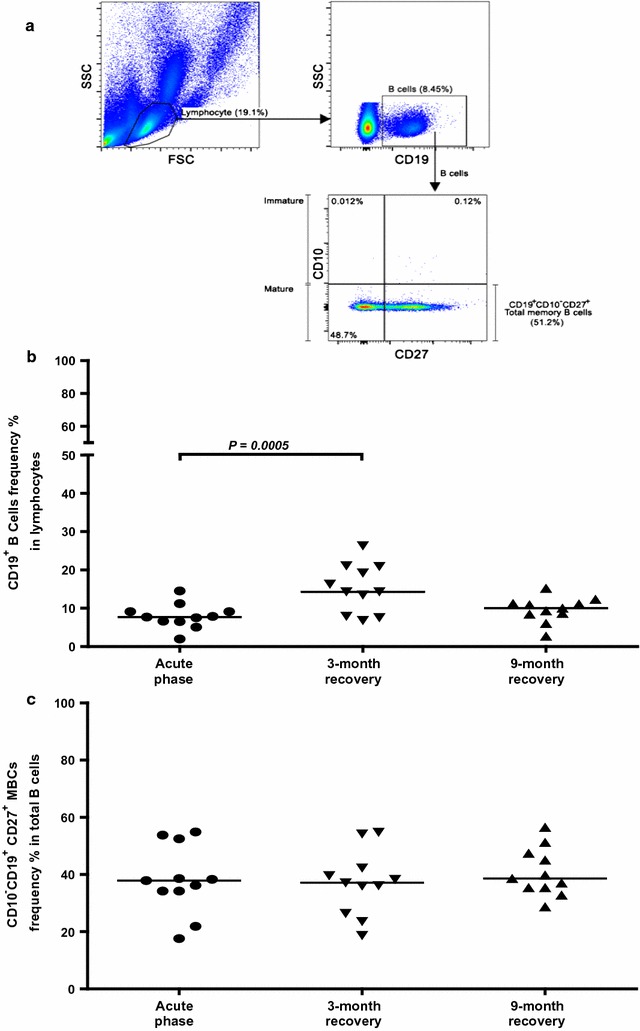
Alteration of CD19^+^ B cells and CD10^−^CD19^+^CD27^+^ memory B cells in *P. vivax* subjects. **a** The gating strategy for identifying the CD19^+^B cells and CD19^+^CD10^−^ CD27^+^ memory B cells (MBCs) by using flow cytometry. The B cell subsets in the FACS *plots* are represented for *P. vivax*-infected patients. The lymphocytes were first gated, and then, 100,000 events using CD19^+^ were collected to identify the B cell populations. These populations were continuously analysed for the expression of CD10^−^ and CD27^+^, which were classified as MBCs. The percentages of CD19^+^ B cells (**b**) and CD19^+^ CD10^−^CD27^+^ MBCs (**c**) in individuals during acute phase and at 3 and 9 months recovery after *P. vivax* infeciton (n = 11) are shown. Each symbol represents the B cell frequencies for one individual. The *horizontal line* reflects the median value

As anti-PvMSP1P-19 antibodies were produced in most of the patients at acute infection, and maintained in some individuals at their recovery phases, the maintenance of total B cells and MBCs were considered for analysis. Here, the frequency of CD19^+^ B cells and CD10^−^CD19^+^CD27^+^ MBCs were demonstrated in *P. vivax*-infected individuals by flow cytometric analysis at the acute phase in comparison with their recovery periods at 3 and 9 months (Fig. 4a). The comparison of CD19^+^ B cells in acutely infected *P. vivax* patients from the same population during the follow-up to their recovery showed a greatly expansion of CD19^+^ B cells at 3 months recovery from infection (*P* = 0.0005, Fig. 4b). Interestingly, the analysis of CD10^−^CD19^+^CD27^+^ MBC numbers in acute infection compared to the convalescence phase showed no significant difference in the MBC numbers (3 months recovery phase; *P* = 0.5985, 9 months recovery phase; *P* = 0.2412) (Fig. 4c).

### Memory B cell response specific to the PvMSP1P-19 antigen after recovery from infection

**Fig. 5:**
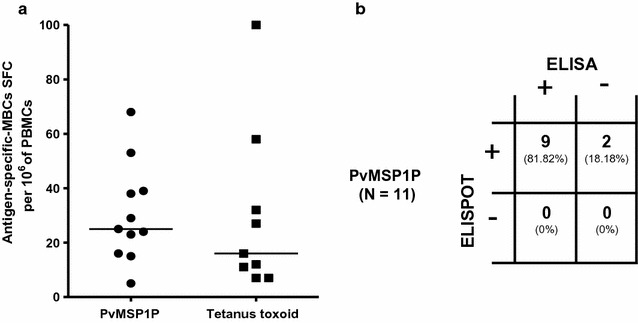
Memory B cell response to the PvMSP1P-19 antigen. **a** The level of the memory B cell (MBC) response to the PvMSP1P-19 antigen and tetanus toxoid in individual PBMCs from 9 months after *P. vivax* infection were determined by ELISPOT (n = 11). The frequencies of MBCs are expressed per million cultured PBMCs. Each *symbol* represents the MBC number for one individual. The *line* reflects the median value. SPM, spots per million. **b** The correlation between the ELISA and ELISPOT responses to the PvMSP1P-19 antigen. The data are shown for 11 subjects at 9 months after recovery from *P. vivax* infection. The numbers of subjects who were double-positive (*top left*), ELISA-positive but ELISPOT-negative (*bottom left*), ELISA-negative but ELISPOT-positive (*top left*) or double negative (*bottom right*) are shown

Because the persistence of CD10^−^CD19^+^CD27^+^ MBCs were detected in individuals at 9 months recovery from infection, the presence of PvMSP1P-19-specific MBCs was demonstrated in the study. PBMC samples from 11 individuals who had recovered from *P. vivax* infection for 9 months were cultured for ELISPOT assays. The analysis of PvMSP1P-19-specific MBCs showed that all the patients (11/11) were positive. The median frequency of PvMSP1P-19-specific MBCs and TT-specific MBCs among responders were 25 and 16 spots per million PBMCs, respectively (Fig. 5a). PvMSP1P-19-specific MBCs were detected in all the samples (11/11) from patients at 9 months recovery from infection, whereas 8 samples (88.9%) were positive for TT-specific MBCs (Fig. 5a). Approximately 81.8% (9/11) of the individuals who were positive for PvMSP1P-19-specific MBCs were seropositive in relation to the antibody titre, whereas 2 samples from seronegative ELISAs were positively detected in PvMSP1P-19-specific MBC responses (Fig. 5b). All the samples showed a correlation between the anti-TT antibody responses and TT-specific MBCs. Moreover, PvMSP1-specific MBCs were also positive at 9 months recovery from infection, at 4–25 spots per million PBMCs. As expected, PvMSP1P-19-specific MBCs were not detected in healthy controls that had no history of exposure to malaria.

## Discussion

The aim of the blood stage malaria vaccine is to limit parasite growth during the stage that occurs within red blood cells and to prevent the clinical symptoms and pathology of malaria. However, most of the vaccines that are currently under development are in a pre-clinical study. The high polymorphisms of many leading candidate blood stage antigens are a major challenge for developing an effective vaccine against diverse strain of the parasites [14, 15]. The PvMSP1 paralog is GPI-anchored protein that is expressed on the merozoite surface, which is involved erythrocyte receptor binding [8]. In comparison with PvMSPs, it has a limited number of polymorphisms and is highly conserved [8]. For the ability in immune stimulation, cellular and antibody mediated immune responses against PvMSP1P-19 was developed in *P. vivax*-exposed patients [10, 11]. The antibody from antisera and *P. vivax* human plasma strongly blocked the binding to human erythrocytes, indicating PvMSP1P-19 antigen contain epitope recognized by inhibitory antibodies. To consider PvMSP1P-19 as a blood-stage vaccine candidate, better understanding long-lived immune response against this protein could be useful for vivax malaria protection.

In this study, the follow up seroprevalence of anti-PvMSP1P responses demonstrated a high prevalence of antibody responses, approximately 72.5, 70.4 and 67.9% at acute phase, 3 months recovery and 9 months recovery phases, respectively. Furthermore, a longitudinal cohort study of anti-PvMSP1P-19 response in 16 individuals at acutely infected phase and after 3-month recovery from infection showed the stability of antibody titres in the absence of reinfection. Interestingly, some patients maintained their antibody response up to 12-month recovery from infection. These data were in line with other studies which showed the maintenance of antibody response to PfMSP1_19_ and PfMSP-1_42_ and PvRAMA after recovery from infection in individuals who were living in low-transmission areas [16–19]. However, in endemic areas with high malaria infection rate had an impact on the efficiency of the antibody response development. Anti-PfMSP1 response was not sustained during the long dry season in Sudan following anti-malarial drug treatment and parasite clearance. Here, the data support the persistence of antibody response to malaria antigen after recovery from infection in low-transmission areas.

Antibody to PvMSP1P-19 had inhibitory activity against erythrocyte binding, approximately 75% of high responders and 30.76% of low responders strongly inhibited PvMSP1P-19 binding to erythrocytes. Importantly, the inhibitory activity was maintained after 12 months recovery from infection in some individuals, suggesting *P. vivax* patients could produce antibodies against PvMSP1P-19 binding to erythrocytes during acute phase, and they can maintain this antibody after recovering from the infection. With regard to the association between the in vitro inhibition assay of antibodies and the clinical protection in malaria infection, the cohort study had been demonstrated in PvDBP vaccine candidates. The maintenance of naturally acquired inhibitory antibody responses decreased the risk of *P. vivax* malaria in rural Amazonians [20] and conferred protection from blood stage *P. vivax* infection [21]. Here, the functional assay of anti-PvMSP1P-19 inhibitory antibody was well demonstrated, further study is required to demonstrate the association between stability of inhibitory antibody responses and its protection from *P. vivax* malaria.

The association between long-lived anti-PvMSP1P-19 responses and the persistence of peripheral CD19^+^ B cells after recovery from infection was observed. However, during acute phase, the total CD19^+^ B cells were reduced compared to non-malaria naive and during convalescence. This data was consistent with previous findings of *P. falciparum* infection study [22, 23]. The reduction of total CD19^+^ B cells could be explained by the temporary reallocation of lymphocytes from the peripheral circulation to the tissues or lymphoid organs following infection [24], or it might occur through the evasive mechanism of *Plasmodium* upon the disruption of B cell lymphopoiesis and the induction of B cell apoptosis as reported in a mouse model [25, 26]. In addition, the expansion of CD19^+^ B cell frequency at 3-month recovery phase may explain the homeostatic regulation of immune system after parasite clearance as there was no significant difference of CD19^+^ B cells population between 3-month recovery from infection and malaria naive individuals, and the expansion of CD19^+^ B cell was not correlated with antibody levels in this recovery phase.

After recovery from *P. vivax* infection, the maintenance of CD10^−^CD19^+^CD27^+^ MBCs was associated with positive PvMSP1P-19-specific MBC responses and circulating antibody titre. However, the positive correlation between peripheral CD10^−^CD19^+^CD27^+^ MBCs and the number of positive spots for PvMSP1P-specific MBC response was not observed. These data suggest that the natural infection could induce PvMSP1P-19-specific MBC development, and those MBCs are able to differentiate among antibody-secreting cells to maintain the long-term antibody responses to this antigens in the absence of reinfection as parasitaemia at 3 and 9 months recovery from infection showed negative by nested PCR. However, the persistence of specific MBCs to the malaria antigens is still unclear. In low-transmission areas, MBCs specific to *P. falciparum* MSP-1 antigen and the *P. vivax* antigens, AMA1 and MSP1-19 was retained after recovery from infection [16, 17, 27]. By contrast, some data from high-malaria-transmission regions showed the expansion of *P. falciparum* AMA1- and MSP1-specific MBCs in acute infection, and then memory cells were contracted to a point slightly higher than pre-infection levels after 6 months of decreased *P. falciparum* exposure [28]. Here, the persistence of PvMSP1P-19-specific MBC response after recovery from infection supports the hypothesis that MBC responses are more stable in low-malaria-transmission areas. In addition, the positive PvMSP1P-19-specific MBC response but negative for antibody titre in 2 adult children suggests that immunity to PvMSP1P-19 can develop persisting MBC responses in some individuals since the initial infection of malaria.

## Conclusions

These results demonstrated that the PvMSP1P antigen is immunogenic to induce humoral response in nature *P. vivax* infection and these PvMSP1P antibodies could be maintained for 9 months after recovery from infection. Moreover, the *P. vivax*-infected patients could produce antibodies to inhibit PvMSP1P-19 binding to human erythrocytes during their acute infection. Those antibodies could sustain strong inhibition until 9 months after recovery from infection. The maintenance of antibody responses to PvMSP1P-19 was associated with the response of MBCs at 9 months after recovery from infection. A greater understanding of the association between the protective immunity and the longevity of MBCs as well as plasma cells will be advantageous for future blood stage vaccine development against vivax infection.

## Abbreviations

PvMSP1P-19: *Plasmodium vivax* merozoite surface protein 1 paralog
MBCs: memory B cells
EBIA: erythrocyte binding inhibition assay
PBMCs: peripheral blood mononuclear cells
CIDR1a: cysteine-rich interdomain region 1a
AMA1: apical membrane antigen 1

## References

[1] Autino B, Noris A, Russo R, Castelli F (2012). Epidemiology of malaria in endemic areas. Mediterr J Hematol Infect Dis.

[2] Hutchinson RA, Lindsay SW (2006). Malaria and deaths in the English marshes. Lancet.

[3] Vogel G (2013). The forgotten malaria. Science.

[4] Gething PW, Elyazar IR, Moyes CL, Smith DL, Battle KE, Guerra CA (2012). A long neglected world malaria map: *Plasmodium vivax* endemicity in 2010. PLoS Negl Trop Dis.

[5] Anstey NM, Russell B, Yeo TW, Price RN (2009). The pathophysiology of vivax malaria. Trends Parasitol.

[6] Herrera S, Corradin G, Arevalo-Herrera M (2007). An update on the search for a *Plasmodium vivax* vaccine. Trends Parasitol.

[7] Riccio EK, Totino PR, Pratt-Riccio LR, Ennes-Vidal V, Soares IS, Rodrigues MM (2013). Cellular and humoral immune responses against the *Plasmodium vivax* MSP-1_19_ malaria vaccine candidate in individuals living in an endemic area in north-eastern Amazon region of Brazil. Malar J.

[8] Wang Y, Kaneko O, Sattabongkot J, Chen JH, Lu F, Chai JY (2011). Genetic polymorphism of *Plasmodium vivax* msp1p, a paralog of merozoite surface protein 1, from worldwide isolates. Am J Trop Med Hyg.

[9] Bozdech Z, Mok S, Hu G, Imwong M, Jaidee A, Russell B (2008). The transcriptome of *Plasmodium vivax* reveals divergence and diversity of transcriptional regulation in malaria parasites. Proc Natl Acad Sci USA.

[10] Cheng Y, Wang Y, Ito D, Kong DH, Ha KS, Chen JH (2013). The *Plasmodium vivax* merozoite surface protein 1 paralog is a novel erythrocyte-binding ligand of *P. vivax*. Infect Immun.

[11] Changrob S, Leepiyasakulchai C, Tsuboi T, Cheng Y, Lim CS, Chootong P (2015). Naturally-acquired cellular immune response against *Plasmodium vivax* merozoite surface protein-1 paralog antigen. Malar J.

[12] Chen JH, Jung JW, Wang Y, Ha KS, Lu F, Lim CS (2010). Immunoproteomics profiling of blood stage *Plasmodium vivax* infection by high-throughput screening assays. J Proteome Res.

[13] Tsuboi T, Takeo S, Iriko H, Jin L, Tsuchimochi M, Matsuda S (2008). Wheat germ cell-free system-based production of malaria proteins for discovery of novel vaccine candidates. Infect Immun.

[14] VanBuskirk KM, Cole-Tobian JL, Baisor M, Sevova ES, Bockarie M, King CL (2004). Antigenic drift in the ligand domain of *Plasmodium vivax* duffy binding protein confers resistance to inhibitory antibodies. J Infect Dis.

[15] Remarque EJ, Roestenberg M, Younis S, Walraven V, van der Werff N, Faber BW (2012). Humoral immune responses to a single allele PfAMA1 vaccine in healthy malaria-naive adults. PLoS ONE.

[16] Clark EH, Silva CJ, Weiss GE, Li S, Padilla C, Crompton PD (2012). *Plasmodium falciparum* malaria in the Peruvian Amazon, a region of low transmission, is associated with immunologic memory. Infect Immun.

[17] Ayieko C, Maue AC, Jura WG, Noland GS, Ayodo G, Rochford R (2013). Changes in B cell populations and merozoite surface protein-1-specific memory B cell responses after prolonged absence of detectable *P. falciparum* infection. PLoS ONE.

[18] Changrob S, Wang B, Han JH, Lee SK, Nyunt MH, Lim CS (2016). Naturally-acquired immune response against *Plasmodium vivax* rhoptry-associated membrane antigen. PLoS ONE.

[19] Torres KJ, Clark EH, Hernandez JN, Soto-Cornejo KE, Gamboa D, Branch OH (2008). Antibody response dynamics to the *Plasmodium falciparum* conserved vaccine candidate antigen, merozoite surface protein-1 C-terminal 19kD (MSP1-19kD), in Peruvians exposed to hypoendemic malaria transmission. Malar J.

[20] Nicolete VC, Frischmann S, Barbosa S, King CL, Ferreira MU (2016). Naturally acquired binding-inhibitory antibodies to *Plasmodium vivax* duffy binding protein and clinical immunity to malaria in rural Amazonians. J Infect Dis.

[21] King CL, Michon P, Shakri AR, Marcotty A, Stanisic D, Zimmerman PA (2008). Naturally acquired Duffy-binding protein-specific binding inhibitory antibodies confer protection from blood-stage *Plasmodium vivax* infection. Proc Natl Acad Sci USA.

[22] Kassa D, Petros B, Mesele T, Hailu E, Wolday D (2006). Characterization of peripheral blood lymphocyte subsets in patients with acute *Plasmodium falciparum* and *P. vivax* malaria infections at Wonji Sugar Estate, Ethiopia. Clin Vaccine Immunol.

[23] Asito AS, Moormann AM, Kiprotich C, Ng’ang’a ZW, Ploutz-Snyder R, Rochford R (2008). Alterations on peripheral B cell subsets following an acute uncomplicated clinical malaria infection in children. Malar J.

[24] Zijlstra EE, el-Hassan AM, Ismael A, Ghalib HW (1994). Endemic kala-azar in eastern Sudan: a longitudinal study on the incidence of clinical and subclinical infection and post-kala-azar dermal leishmaniasis. Am J Trop Med Hyg.

[25] Matsumoto J, Kawai S, Terao K, Kirinoki M, Yasutomi Y, Aikawa M (2000). Malaria infection induces rapid elevation of the soluble Fas ligand level in serum and subsequent T lymphocytopenia: possible factors responsible for the differences in susceptibility of two species of Macaca monkeys to *Plasmodium coatneyi* infection. Infect Immun.

[26] Bockstal V, Geurts N, Magez S (2011). Acute disruption of bone marrow B lymphopoiesis and apoptosis of transitional and marginal zone B cells in the spleen following a blood-stage *Plasmodium chabaudi* infection in mice. J Parasitol Res.

[27] Wipasa J, Suphavilai C, Okell LC, Cook J, Corran PH, Thaikla K (2010). Long-lived antibody and B cell memory responses to the human malaria parasites, *Plasmodium falciparum* and *Plasmodium vivax*. PLoS Pathog.

[28] Weiss GE, Traore B, Kayentao K, Ongoiba A, Doumbo S, Doumtabe D (2010). The *Plasmodium falciparum*-specific human memory B cell compartment expands gradually with repeated malaria infections. PLoS Pathog.

